# An Account of Putrid Sore Throat, as It Occurred in Some Parts of Ohio

**Published:** 1845-02

**Authors:** John Dawson

**Affiliations:** Jamestown, Ohio


### WESTERN JOURNAL

OF

### MEDICINE AND SURGERY.

FEBRUARY, 1845.

Art. I.—*An Account of Putrid Sore Throat, as it occurred in some parts of Ohio.* By John Dawson, M.D., of Jamestown, Ohio.

The design of this paper is to give some account of *Putrid Sore Throat* as it has manifested itself in our region for the last three or four years. Hippocrates says: "*Past things must be learned, present known, and things future foretold.*" Uttered as this has doubtless been, in reference to the duties devolving upon every physician; the mandate nevertheless, while it points out what ought to be done, makes rather too heavy a draught upon our feeble powers.

Of the '*past things*' we may attempt to give some account, but of the '*present*,' we always know too little, and of the '*future*,' generally nothing at all.

So far as the disease which is the subject of this essay is concerned, we could heartily wish the mandate of the 'Father of Medicine' capable of being obeyed. We should be glad to have learned everything of any consequence in relation to the history of *putrid sore throat*, or to be able to know exactly what it is *now*, but we should be still more gratified, if we were able to tell what its associations, or morbid phases, or even its results in any single case, may be in future.

Unable, therefore, to comply with any part of the injunction, we nevertheless look upon it as being an epitome, or rather the archetype of that system of philosophy denominated the *inductive:* viz. The observation of facts and the embodying of those conclusions that legitimately flow from them; and we suppose that the time will come, when the Father of the medical profession, at any rate, will be regarded as a fellow-laborer in building up the inductive system. But more immediately to our subject. We shall give some facts in regard to the prevalence of *putrid sore throat*, and then make such inferences as seem to be warranted.

The number of cases, which have fallen under our notice within the last three or four years, has been twenty-two.

Eleven of these occurred to children under ten years of age; four to children between ten and sixteen; and seven to adults. The youngest child was one year old, and the oldest adult was over sixty.

So far as sex is concerned, I have observed no difference, though my cases have not been sufficiently numerous to test this matter with accuracy.

Predisposed the most to this disease, have been children of a weak lax habit, or those laboring under a phthisical, or scrofulous diathesis. Generally, too, was noticed a want of that attention to cleanliness and clothing, so necessary to any thing like health. Four of the adults were of feeble constitution, and health more or less infirm. In the remaining three no constitutional disturbance of any kind was observed.

Most of the cases occurred after the breaking up of the winter and during the spring months, March, April, and May. Occasionally a case took place after this time, or during the cold damp weather of winter. These, however, were rare, the disease having displayed, so far as our observations have extended, a decided preference for the cold, damp and variable weather of the spring months. The past winter and spring, in our region, having been characterized by much rain, rapid changes in the temperature of the atmosphere, and a low range of the thermometer, we have had as a consequence more disorder of the throat than usual.

The number of families in which these cases occurred, was eleven, averaging two cases to the family; and the greatest number affected in any one family was four.

After its occurrence in a family, it was seldom noticed to spread to all the members, one or two generally being affected, while the rest would escape. In two instances the mother contracted the disease after the children had commenced convalescence.

HISTORY OF THE DISEASE.

By some name or other this disease has been known since the time of Hippocrates. The '*paristhemia*' of this author, signifying a throat affection, although too general to convey any thing like a correct idea of the lesions which usually occur in a case of putrid sore throat, is however as definite in meaning as the terms, '*squinsy*,' '*quinsy*,' '*cynanche*,' or '*angina*,' as used by later, and some modern writers, to designate inflammatory affections of the mucous membrane lining the palate and pharynx, and covering the tonsils.

It has been supposed that the '*Syrian* and *Egyptian ulcers*' mentioned by *Areteus Cappadox*, and the '*pestilent ulcerated tonsils*' of *Etius Amidenus*, were of the character of putrid sore throat. Be this as it may, it seems pretty certain that the *malignant ulcers* of the *mouth and jaws*, described by *Riverius*, the *malignant squinsy* of *Brookes* and *Ball*, the *gangrenous quinsy* of *Böerhaave*, the *epidemic quinsy* of *Swan*, the *angina maligna* or *ulcerous sore throat* of *Huxham* and *Fothergill*, and the *chynanche maligna* of *Cullen*, and some other writers, refer to the same disease. Indeed the accounts, although they are in many instances very brief and imperfect, consisting sometimes in nothing more than a bare mention of the disease, are, nevertheless, in some English as well as continental writers anterior to the present century, detailed with considerable accuracy. I allude to the essays of Fothergill and Huxham, both of whom are entitled to the credit of having brought before the profession much of what is now known respecting this malady. To Huxham in particular we are indebted for a faithful history of the disease as it prevailed in his town and other parts of the British dominion during the years 1751–'2–'3. Seemingly from the account of this very ingenious author, the disease was considered as having an identity of its own, as being different in history, symptoms, and the effects of remedies from all other diseases, but as bearing a close relation, in some few particulars, to typhus fever. Since Huxham's time the confusion of nomenclature has, as in ancient times, enveloped the subject; and we have Cullen and Thomas considering it as a variety of *quinsy* or cynance, (*cynanche maligna*), Rush, McIntosh, Eberle, and their cotemporaries as a variety of scarlatina, (s. maligna), and Bretonneau as a variety of croup, (diptheritis). Without much doubt there is something wrong in these classifications, and in another place this matter will be more fully discussed.

### CAUSES, REMOTE AND PROXIMATE.

Unable as we are to tell anything about the specific virus by which many of our epidemics and endemic diseases are originated, we may, notwithstanding, entertain some hope that these mysterious agents may yet be brought to light by closely and assiduously cultivating every fact connected with their origin, progress, and termination. The disease we are considering, like many others, evidently has some relation, so far as its origin is concerned, with the season of the year, locality, habits, and thermometrical and barometrical conditions of the atmosphere. Invoked faithfully wherever this disease prevails, these circumstances may yet untie the 'gordian knot.' At any rate, they may always be regarded as essential to success in the treatment.

As early as the time of Hippocrates this disease was observed to prevail most in the "winter and spring time, when slimy phlegm falls from the head to the jugular veins which obstructeth the passages of its spirits with its cold glewiness"—(Riverius). In his '*Dissertation on Ulcerous Sore Throat*,' Huxham found it to prevail most during the fall and winter, that had been preceded by a cold wet summer, (1751) in which a "vast quantity of rain fell. The summer was in general uncommonly wet, cold, and frequently stormy.—At the beginning of June, however, we had exceedingly hot weather, and some very sultry days in July and August; the atmosphere was almost always thick and moist, but the barometer commonly low. The fruits of the earth were crude, watery, and insipid, the harvest was excessively bad, and the grain of all kinds suffered greatly." Our author adds, that although at this particular time there was not much sickness prevailing, yet "there were hysterical and hypochondriacal diseases to some considerable extent; and there were also a kind of universal inactivity and lowness of spirits every where, and in all sorts of fever there was a surprising disposition to eruptions of some kind or other, to sweats, soreness of the throat, and aphthæ." Putrid sore throat, says Thomas, "often arises from a peculiar or humid state of the atmosphere, and so becomes epidemical, making its attacks chiefly on children and those of a weak, lax habit, principally about autumn and the beginning of winter." F. P. Emangard states, (in a *Memoir on Epidemic Angina*, *Paris*, 1829), that he had an opportunity in the year 1828 to observe some of the circumstances connected with the origin of this disease. "That year was characterized," says our author, "with frequent and abundant rains. The portion of the country where this epidemic presented itself is cut up by dales, at the bottom of which there flow small streams subject to inundations, and is well covered with underwood, fir-trees, and forests, combining all the causes of permanent humidity, and a temperature nearly always cold. The inhabitants are located in these dales, on the borders of the woods, or in the midst of orchards, so as to be nearly deprived of the beneficent influence of the sun, and at the same time exposed to the action of the marshy exhalations." Here an epidemic angina maligna made its appearance towards the end of February, 1828, and continued until autumn.

But few cases, as has already been remarked, of this disease took place in our region anterior to the past winter and spring, and these, so far as could be observed, were in persons of a weak, lax habit, generally children, laboring under a diathesis, either phthisical or scrofulous, or chachectic from bad diet, a want of clothing, or the necessary attention to lodging and cleanliness. The month of October last, was ushered in with more rain than usual; and this continued to be a characteristic of the weather during the balance of the fall and winter. Indeed, the winter was unusually open, the weather variable, and so great an amount of rain had fallen that when the wet weather of March, April, and May came on, many sections of the country, embracing generally the flat table lands, were more or less inundated; and they continued in this situation until June, and in some instances until July. As a consequence of this state of things, thoracic diseases, pneumonia, pleuritis, &c., which are most common to the spring months were scarce, and in the majority of cases mild. Typhus fever, the "*black-tongue*," and the anginas, seemed to have taken their place, and constituted the principal disorders, in connexion with parotitis and measles, both of which were epidemic during the spring months, that we were called upon to treat. The angina attained nothing like a general prevalence. Confined almost exclusively to certain neighborhoods, it constituted almost entirely the only disease with which they were visited, during the season. Particularly, we did not rate the prevailing winds, but Riverius says, upon the authority of Hippocrates, that the angina of ancient times prevailed most during the presence of '*southerly winds.*' The thermometrical condition of the atmosphere in our vicinity, was characterized by great variations. For a few days the weather would be warm, with a moist atmosphere, imparting to plants the first impulses of vegetation, when suddenly a cold north-west wind would make its appearance and blight the prospect of returning spring. This state of things continued through March, April, and a good part of May. Nothing more in regard to the etiology of the disease was observed from these vicissitudes, than what might naturally have been expected, an aggravation of the symptoms when the change was from hot to cold. Dr. Green, of Bainbridge, Ohio, who happened in my office while engaged in writing this essay, says that he has observed putrid sore throat to prevail in his vicinity during the fall and spring, when the weather was cold and damp, that he saw the most cases along the bottom lands of Paint creek, and some few in the 'beach flats' in the vicinity of Bainbridge.

Concerning the sex most liable to this complaint, the opinions are various. *Hippocrates*, in describing an *Epidemical Constitution*, in which anginas, coughs, and pluro-pneumonias were frequent, says that men were most liable to angina, and the reason he gives is, "because they were more abroad than women, and therefore more subject to injuries of the air." *Riverius* thinks also that men are the most subject, and gives as a reason of this, that "women have a *colder blood*, a *less larynx*, and narrower veins of the throat, for which reason these parts do not easily receive defluxions." Modern writers have observed sometimes a preponderance in favor of males; and at other times in favor of females, making, as we believe, if the accounts were all compared, no difference as it regards predisposition to the complaint. We may now sum up the following circumstances as favorable to the prevalence of this malady:

1. The cold, humid weather of the fall and spring.

2. Localities characterized by a low range of the barometer.
3. Habits of the weak effeminate kind, with some constitutional derangement of the system.
4. 'The springs of life,' that development of the nervous system found in children.

These having characterized its prevalence in past times, may be confidently expected to have some relation with it in the future.

Into a consideration of the *contagious* character of this disease we do not design to enter, further than to remark, that Huxham regarded it as being highly contagious; and from what we witnessed of its prevalence it seemed to have a close resemblance in its origin and propagation to typhus fever. One thing is very certain, that the peculiar virus, by which it is communicated from one individual to another, displays a striking preference for children; and where it does invade the system of an adult, its effects are generally mild. That severe cases attended with much putridity may elaborate a virus adequate to produce the disorder in others, much exposed to its influence, seems pretty evident from analogy, as well as from the fact, that the two mothers, to whom allusion has heretofore been made, contracted the disorder after having waited upon their children.

As it regards the proper pathology of this disease, physicians have always been much perplexed. While some seem to think all the lesions due to a fever of the typhoid kind, others there are that regard it as a local affection, either of the skin, mucous membranes generally, or of the mucous membrane lining the mouth, and consider the febrile orgasm as be-being exclusively symptomatic. This diversity of opinion grows, in part, out of the insidious and obscure manner in which many cases are invaded by the remote cause. Not universally, but commonly the cases falling under our notice have been ushered in with the premonitory symptoms of fever, such as languor, muscular debility, cephalalgia, aching of the bones, and chilliness. To these a fever very much like the typhus mitior of Cullen succeeded. The febrile excitement in adults was slight. In children, on the contrary, it was sometimes violent; and, in both adults and children it always appeared to be proportionate to the structural changes in the throat which accompanied the disease, or with which it was complicated. The pulse in the adults was soft, frequent occasionally, but in no case indicated the use of the lancet. In the children it was, with few exceptions, frequent, small, and sometimes very irritable. The tongue generally was foul.

PHYSICAL CONDITION OF THE GENERAL SURFACE.

The functions of the skin were more or less deranged in all the cases we witnessed. Sometimes dry when the febrile excitement was considerable, at other times bathed with a clammy offensive perspiration, *but in no case covered with an eruption.* This negative symptom, I am aware, does not correspond with the observations of the generality of writers. In his *Dissertation* on this affection, Huxham says, some had a cuticular eruption, others had none; sometimes the eruption was of the pustular, then again of the erysipelatous kind. Ball, in speaking of this disease, says, it was occasionally characterized by an erysipelatous eruption on various parts of the body. Neither Sydenham, Boerhaave, nor Van Sueiten gives any account of eruptions attending the quinsies or anginas of which they speak. Swan, Sydenham's commentator, however, describes an *external* species of quinsy, which "extended towards the eyes, and chiefly possessed the external muscular and glandular parts." Thomas says, "there was a dark colored eruption in some cases, which is an unfavorable symptom." Eberle says the eruption comes out at uncertain periods, from the second to the fourth day, and is usually pale when it first makes its appearance, acquiring in most instances a dark or livid hue. Cullen witnessed an eruption after the disease had progressed for a few days. Gregory and McIntosh observed eruptions on the skin in this disease; and both, regarding the efflorescence which takes place in all eruptive fevers, as a kind of blistering process instituted by the *vis medicatrix naturæ* for the relief of these disorders, considered the fatality attending many cases to be due to a want of appearance of the eruption. Dr. Warfield, of Paris, Kentucky, describes an epidemic angina, (in the first volume of the Western Journal) as it prevailed in his vicinity, and says "not a single case was attended with efflorescence." Of the practitioners of medicine in Ohio with whom I have conversed, some have seen the disease prevail with and some without an eruption. From these authorities it appears that the eruption is by no means a constant attendant on this complaint. But what seems most difficult is the estimation we should place upon it, as an element of the pathology. That it is a mere symptom in this affection, as well as in eruptive fevers in general, constituting no part of the pathology of any of them, McIntosh seems very certain. Against this opinion objections might not be very successfully urged. But to make the eruption both a symptom, indicating merely the existence of disease, and also a means instituted by nature for the relief of the disease, seems to us like confounding symptoms and remedies together. Without pretending to criticise Dr. McIntosh on this subject, it may be remarked, that among most writers there seems to be a supposition that the eruption, some how or other, is connected with the pathology; and when it fails to make its appearance they regard the equilibrium of the disease as being destroyed; and imagine that all the distress following a bad case proceeds from congestions, and the centripetal direction of the fluids to the deep-seated organs of the body: hence the efforts usually made with the warm bath, frictions, etc., to invite the excitement to the surface. These views, no less than those of Dr. McIntosh, become, so far as *angina maligna* is concerned, very much unsettled, from the fact that many of the mildest cases of the complaint are unattended with any eruption whatever. And, even in those where the eruption does actually exist, it is so diversified in character and extent, resembling at times erysipelas, petechia, or erythema, that its appearance or disappearance, unlike what generally takes place in measles or scarlatina, is accompanied by no very obvious modification of the malady. From these considerations we infer, 1. that the disease may prevail accompanied with eruptions of various kinds, or it may prevail without any at all; 2. that the eruption bears, when present, about the same relation to the disease that the petechia, sudamina, etc., do to typhus fever.

In the anatomical condition of the stomato-pharyngian mucous membrane, is, perhaps, exhibited the most important elements in the pathology of this disease. Upon this point the enemy concentrates and marshals its strength, not only for destruction here, but for the invasion of other parts of the body. Not many days usually elapsed after the first sensations of indisposition until the throat became the seat of pain, burning sensations and unnatural dryness. In some instances the disease first manifested itself in the throat. Inspection of the parts, soon after the patient would commence complaining, showed the mouth and fauces to be affected with various degrees of hyperemia or inflammation. The mucous membrane, in a few cases, presented rather a bright red appearance. But in the majority it was *dark* from the commencement, and seemed most intense where it covered the velum pendulum palati, the uvula, and tonsils. After lasting a few days in the mere form of palatitis, uvulitis, or tonsilitis, the urgency of the symptoms in a few cases, principally adults, subsided without any obvious alterations in the structure of the mucous membrane. It was, however, very liable to return upon physical exertions, which I found to be a powerful, and very common exciting cause; or imprudent exposure to bad weather, cold, damp air, &c. Cases of this kind were more or less subject to relapses for months after they had contracted the malady; but, contrary to what might *a priori* have been expected, the relapses were attended with no increase in the malignity of the symptoms. In some cases of children where the disease was ushered in by even considerable febrile excitement, the fauces assumed that tumefied dark red aspect, termed in former times "*defluxions*," but in our day passive inflammation, or asthenic hyperemia. The presence of too much venous, and the seeming absence of all arterial blood in the parts is perhaps what gives rise to this condition. Continuing, for a longer or shorter time, to increase in darkness and tumefaction, the integrity of the mucous membrane at last became assailed in a way that ultimated in gangrene and the death of the parts most affected, giving rise to ulcers on the tonsils, velum pendulum palati, or uvula. In some instances the ulcerative process, in spite of remedies, continued until it had eaten off large portions of the soft parts in the fauces. These ulcers generally first made their appearance on the mucous membrane reflected over the tonsils, and extended from here to the velum and uvula, and sometimes into the larynx, giving rise to dysphagia and partial aphonia. I saw several children afflicted with this form of the disease in whom the swelling or hypertrophy of the tonsils and glands on the side of the neck was so great as nearly to close up the air-passages. Upon the tonsils and contiguous parts were frequently observed small patches of an ash-colored false membrane, under which was sometimes concealed the commencement of these malignant ulcers. These cases pretty generally went to a fatal issue from suffocation. The fatal event was sometimes preceded with restlessness, anxious expression of the countenance, a livid color of the lips and more or less ronchus. Other cases, however, died very suddenly without betraying any suffering at all in the last moments. One child, after having conversed with its mother, turned over in the bed, and in a few seconds was dead. Two others who were possessed of sufficient muscular energy to stand up at the table while the family were at dinner died in the evening of the same day, with but slight distress in the function of respiration. The fatality of these cases, inasmuch as the secretion of false membrane was inconsiderable, doubtless depended upon the ulcerative and gangrenous processes extending into the larynx, trachea, œsophagus, and contiguous cellular tissue. Dr. Morris, in the *Quarterly Summary of the College of Physicians of Philadelphia*, gives a case in which the symptoms were somewhat similar to those we have described. In this case the *sectio cadaveris* revealed an abscess, situated between the œsophagus and vertebra, which contained about half an ounce of purulent matter. Dr. Ballot, physician to the Hospital of Gien, reports an analogous case. On the anterior face of the vertebral column, and in contact with the posterior coat of the œsophagus, was found a collection of white well-concocted pus. Other cases of a similar nature have been referred to by different writers, under the name of *retro-pharyngeal* or *retro-œsophageal abscess.* To structural alterations of this kind, are due many of those cases of ulcerous sore-throat, which have an obscure yet fatal termination, and in which, during life, there is nothing discoverable to which such a grave and sudden issue could, with any reason, be ascribed.

The most interesting, because the most fatal form of this disease as it manifested itself in the fauces has yet to be described. The commencement and progress differ in no very material manner from the other forms to which we have adverted. There is likely more fever, and the fauces in the forming stage of the malady are of a brighter color. At uncertain periods in the progress, sometimes after the third or fourth day, or even later, there is thrown out upon the stomato-pharyngean mucous membrane a membraniform exudation, which, showing itself first upon the tonsils, gradually extends over the fauces and sometimes to the air-passages, œsophagus, nasal fossæ, external auditory duct, or the temporal surface of the concha. "The first symptom we observe," says Andral, "is a number of red dots or streaks scattered over the surface of the mucous membrane, which does not in general present any remarkable degree of tumefaction. Sometimes, however, from the very onset of the disease the surrounding cellular tissue is congested, and the submaxillary lymphatic ganglions are considerably swelled. The red appearance of the membrane is, after a longer or shorter interval, succeeded by a set of white spots, which are at first isolated and seem to exist chiefly in the follicles, but afterwards multiply, enlarge, touch, and at last run together, so as to form a uniform layer of greater or less extent. Sometimes there are several patches; and sometimes there is only one, which covers a vast space and is continually extending itself. The thickness of this layer is variable; it is occasionally thick enough to be in some degree transparent. One of its surfaces is free; the other, which adheres to the mucous membrane, presents a great many processes which dip into the mucous follicles. Its color is generally white, but it is sometimes grayish and soiled by the blood exhaled by the mucous membrane which gives to it an ashy tint; that, together with the extreme fetor of the secretion, has often caused such patches to be taken for sloughs of the mucous membrane. At other times patches of small extent, and lying lower than the surrounding mucous membrane, have been mistaken for ulcers." To this form of the disease modern writers, upon the supposition that it was a distinct disease, have applied various names, as *angina membranacea*, *angina pultacea*, or *casei formis*, (*Guersent*,) or *diptheritis*, (*Bretonneau.*)

In the refinements of medical nomenclature many might suppose that these terms indicated, not merely a form or variety of putrid sore-throat, but a distinct disease, having an identity of its own, and demanding for its relief a corresponding difference in the treatment. Apprehensive as we are that the former, much more than the latter, of these suppositions is not founded upon a correct consideration of the different morbid phases assumed by putrid sore-throat, we shall not, however, insist upon this point at the present, but shall remark, that while we witnessed more or less of this membranous exudation in the fauces of the majority of our patients, there were four of the twenty-two, to whom we have previously referred, in whom it extended into the larynx, producing *secondary croup*, or the *diptheritis* of Bretonneau. These four cases were all under ten years of age; and among the best in constitution and habit of any of the cases we witnessed. The anginose symptoms were tolerably urgent from the beginning, though I think without any ulceration of the fauces.

There were more fever and restlessness than usual after the signs of distress in the larynx were manifested. Coming on very insidiously, the exact period from the commencement of the disease at which the secretion in the larynx took place, could not be ascertained. Perhaps it was not until four or five days had elapsed; and in two cases there was no unusual trouble in the larynx for two weeks. Seated once in the larynx the circulation, sooner or later, becomes frequent and irritable, the face flushed, and the eyes suffused, with the cough and respiration peculiar to croup. The treatment was varied in all these cases so as to suit the indication, but they all died; and this, so far as we have learned, has generally been the issue of similar cases in the hands of other practitioners. Indeed, when the disease takes this turn, it may be looked upon as being necessarily fatal, since nothing but an accidental expulsion of the membrane, or an operation upon the trachea, could be looked upon as promising a *chance* of escape; and when we consider the extreme improbability of the one, and the uncertainty in the results of the other, all hope is entirely diminished. We shall now, therefore, briefly consider some of the circumstances connected with the secretion of this morbid production.

Properly considered the appearance of false membrane in any part of the body must be looked upon as a *lesion of secretion*, as the product of an organic action more or less analogous to that, which, in the healthy state, separates from the blood the materials for repairing the several tissues or forming the different secretions, (Andral.) And according to elementary writers on pathology, the secretion of false membrane is more or less influenced, 1. by *the state of the blood*; 2. *by the condition of the solids, where the secretion takes place; and* 3. *by the nervous system.* Huxham says that he found the blood so soft and loose that you might cut it with a feather, giving off little or no serum. In some cases the blood first drawn by this author was covered with a "*thin whitish*, or *lead-colored skin;* but under this there was a *greenish soft kind of jelly*, and at the bottom a very *loose black kind of crassa-*

*mentum* scarcely at all cohering." A thin dissolved and incoagulable blood seemed to be characteristic of the disease in former times; but whether this is the condition of the blood most favorable to the secretion of false membranes, the chief constituent of which appears to be *fibrine*, is a question that must be settled by future research. The "*thin lead-colored skin*" observed by Huxham on the blood of some of his patients owed its existence, no doubt, to the same cause which gives rise to the buffy coat in chlorotic patients, viz: excess of fibrine in proportion to the number of globules. Why is it that this fibrine is secreted from the blood and made to assume, on parts in a state of irritation, the form of false membrane? Is it that the relative constituents of the blood may become balanced, so as to approximate to something like the normal standard? About all we know concerning the condition of the solids, before and while the secretion of false membrane is going on, is that the parts must be in a state of irritation, or hyperemia. Formerly it was supposed that the ash-colored patches observed in the fauces, were a kind of slough proceeding from an ulcerative process. This, however, is not the case, for in most of the cases where false membrane makes its appearance the integrity of the parts beneath it seems preserved. Indeed, the membrane in some cases may be peeled off, leaving the mucous surfaces below without any apparent abrasion. The secretion of false membrane, we are aware, does not occur with most facility in those cases in which the fauces assume a very dark red appearance from the beginning, called "*asthenic hyperemia;*" these cases being more disposed to run into gangrene and ulceration. It is a color a shade brighter than these that is most favorable to the secretion.

Of the condition of the nervous system most favorable to the development of false membranes on the mucous surfaces, we know little or nothing. There is in most cases of putrid sore-throat, before and after the appearance of false membranes, an adynamic disposition of the general system similar to what we have in typhus fever. This perhaps should be regarded as the result of a lesion of innervation, in which the nervous forces, from some influence exerted upon them by the remote cause, cease, in a measure, to exercise that resistance to the affinities of inorganic matter, that are really necessary to preserve the blood in a normal condition, and keep, in severe cases, the local parts most diseased from passing into a state of gangrene and death. With more certainty, however, we know that these alterations occur with most facility during the period of life in which the nervous system is not perfected, but exists in a progressive state of development. The few cases we witnessed, in persons advanced in life, were unattended with any membraniform exudation, either in the fauces or air-passages, while among the children, and to some extent those bordering upon puberty, the phenomenon was by no means unusual; and hence this circumstance, at any rate, may be regarded as having a close relation to the occurrence of this morbid production.

From some considerable research on the portion of the alimentary canal most liable to this secretion, it has been discovered that the supra-diaphragmatic is more frequently affected than the infra-diaphragmatic portion. Of two hundred and fourteen cases of aphthæ observed, in the *Hospital des Enfans-Trouves*, by M. Billard, during the year of 1826, false membranes were found in the stomachs of but three, and in the intestines of but two. M. Lelut, who wrote a work on aphthæ, saw them seldom in the stomach and never in the intestines. The case of a girl twelve years of age is given by Andral, in whom not only the air-passages were lined with false membrane, but they were also discovered in the pharynx, œsophagus, and *stomach*. Aphthæ, although it differs very considerably from putrid sore-throat, may, nevertheless, from some points of analogy, serve partially to illustrate the relative predisposition of the different portions of the alimentary canal in the latter disease to this morbid secretion.

Formerly it was supposed that *albumen* was the chief constituent in the composition of false membranes. Recent research, though, by M. Lassaigne, seems to throw additional light on this interesting subject. "I have already," says he, "shown that the false membranes, which are thrown out on mucous membranes in a high state of inflammation, are not composed of coagulated *albumen*, as many anatomists have supposed; but formed chiefly of a large proportion of *fibrine* mixed with some soluble albumen, and moistened by a yellow-colored serum, containing all the organic and inorganic elements of the blood. I have more recently examined the false membrane of a pig, laboring under pseudo-membranous angina, and obtained the same results. The false membranes obtained from this source were white, with a yellowish-colored tinge, slightly elastic and extensible." On submitting them to pressure a yellowish-colored fluid was obtained which turned test paper blue, and coagulated under the influence of heat and the mineral acids. Having washed a portion of the false membrane in cold water, to remove all the soluble matter, our author found that the residuum was a white substance presenting all the physical and chemical characters of fibrine extracted from the blood. Digested with weak acetic acid it became swollen, then transparent, and on the application of gentle heat was entirely dissolved. When saturated with caustic potassa, the solution threw down white flocci which were again dissolved by an excess of the alkali. The nitric, sulphuric, and muriatic acids, threw down a white precipitate, as they do with the acetic solution of fibrine. Having evaporated the water in which the false membranes were washed, there remained flocci of coagulated albumen; the evaporation being continued until there was dryness, a saline residuum remained, composed of chloride of sodium, carbonate and lactate and phosphate of soda, salts which exist in solution in the serum of the blood.

Formed either on the mucous or serous membranes, the substance of which false membrane is composed is capable of becoming organized. The tendency to organization is much less marked on the mucous than on the serous membranes. In relation to this process the first phenomenon observed is, that the membrane becomes injected with blood. Andral says a question might be started here to know how this fluid gets into its new situation; whether it is absolutely formed in the membrane, or whether it is brought there by vessels which shoot into it from the surrounding tissues. Advocates there are for both of these hypotheses; and from the facts adduced in support of each of them we may conclude, 1. that there is some blood in false membranes not contained in blood vessels at all, but irregularly scattered through their substance in dots or lines; 2. that vessels have been discovered pervading them which do not communicate with those of the surrounding tissues; 3. other vessels have been discovered which seem to proceed from those of the surrounding tissues; or rather the vessels of the parts upon which the false membranes are situated appear to be extended into them. The manner now in which the circulation commences is, that, either in the organizable matter, or in points where it adheres to the membrane, or in that membrane itself, there must begin to move some globules which, after proceeding in various directions, and tracing out passages for themselves, shall end by falling into currents already long established. (*Andral.*)

Besides the presence of blood vessels in false membranes, there are, according to *Prof. Vanderkelk of Utrencht*, very good reasons for believing in the existence also of *lymphatics.* This author refers to a case in which the pleura had been inflamed and had thrown out coagulable lymph, which, by a considerable bridle, connected it with a part of the diaphragm. The surface of this connection was not less than an inch or two in extent, and the bridle varied from a quarter of an inch to an inch in length. The lymphatics of these parts having been injected with quicksilver, the vessels of this system belonging to the lungs and pleura were very conspicuous; not less so those of the diaphragm; and not less apparent than either, were *distinct beaded lymphatics, running along the effused membranes connecting the two normal tissues.*

Nobody, so far as we are acquainted, has yet discovered any nerves in these adventitious membranes. When this is done, and we see no good reason why it should not be, all the prerequisites will be present for an exhibition of the common phenomena of vitality. That nerves do exist in them, might be concluded *a posteriori* from the fact, that the function of circulation, in the present state of our knowledge, stands in the relation of effect to that of innervation; and to us the presence of one necessarily implies the existence of the other. Indeed, without the presence of the nervous influence in these membranes, the vascular system could not be developed, nor could the fluids be circulated through them. Speculative as this may appear, it has nevertheless a degree of probability in its favor, that, sooner or later, we believe, will be made to assume the character of demonstration.

We have been thus prolix in our consideration of these false membranes, because whether regarded in the light of an occasional complication of putrid sore-throat, or as a part of the disease itself, they cannot fail, when present, to excite in the mind of the practitioner the keenest solicitude for the welfare of his patient. And well this may be the case; for from my own limited experience, and what I have gathered from other practitioners of medicine, I conclude, that no small part of the mortality attendant on this truly mortal disease, is referable to the presence of this morbid exudation. And when it is recollected that but few cases occur in which it is not more or less developed in some part or other of the alimentary canal or air-passages; and that it is liable at any stage of the malady to extend to some vital part and produce death, a reason may be seen, why even an elaborate consideration of the subject could not fail to prove interesting.

Before we conclude what we have to say, a brief review of the points insisted upon in the foregoing inquiry will now be submitted.

1. The appearance of false membranes on the mucous surfaces of any part of the body, is the result of an alteration of their secretory process.

2. There is a striking alteration of the blood connected with cases of putrid sore-throat in which false membrane is secreted, consisting, most probably, in a deficiency of globules, which gives rise to its dissolved putrid appearance.

3. The only known condition of the solids upon which the false membrane makes its appearance, is inflammation.

4. In general there is no abrasion of the mucous surfaces from which the membrane takes its rise, though in a few cases ulcers under the false membrane have been discovered.

5. That development of the nervous system found in children, and in those under the age of puberty, seems most favorable to this secretion.

6. The portion of the alimentary canal most predisposed is the supra-diaphragmatic.

7. The chief chemical constituent is *fibrine.*

8. Arteries, veins, and lymphatics have been demonstrated to exist in these adventitious membranes, and they are probably endowed also with nerves.

*Diagnosis.*—Having discussed some of the more important lesions connected with *angina maligna* or putrid sore-throat, we will now institute a brief comparison between it and scarlet fever, that we may see in what respects the two diseases agree. We are induced to do this because some of the more modern writers, including Thomas, Rush, McIntosh, and Eberle, have regarded putrid sore-throat as a mere variety of scarlatina, having no claims whatever to the character of a specific disease; and consequently demanding for its treatment nothing but a modification of that proper for the latter malady. Viewed in several aspects, the two diseases present quite a similarity. 1. They both prevail mostly during the variable weather of fall and spring; 2. to both diseases children are the most liable; 3. both may prevail with an eruption; 4. both are reputed to be more or less contagious; 5. there are anatomical lesions of the throat in both diseases, and each at times is complicated with membraniform concretions. There are points in which, between the two diseases, there seems to be something like a unity of diseased actions. Desirous to avoid making distinctions where there is no difference, we, nevertheless, in order to take an impartial view of this subject, are compelled to a brief consideration of some facts in connection with the origin and progress of these two diseases, which may go, to a greater or less extent, to exhibit a contrast.

1. Although it is mentioned by some writers, Thomas among the rest, that the two diseases are alike, because they prevail together, some children of a family having one form or variety and some the other, we confess, notwithstanding we have witnessed several epidemics of scarlatina, that we have not yet witnessed one of these in which there were any well marked cases of putrid sore-throat. True a great many cases, during the prevalence of an epidemic scarlatina, may have the anginose variety of the disease in a very malignant form; and instances of this kind we have frequently witnessed; but such cases, in our estimation, contrasted very strongly with putrid sore-throat. On the other hand, when we have had cases of sore-throat occurring, either sporadically or epidemically, no scarlet fever, so far as we ever knew, was prevailing in the neighborhood at the time.

2. Adults, as a general rule, escape scarlatina. At any rate we are acquainted with no instance in which, as an epidemic, it displayed towards them anything like a total preference. In the epidemics of membranous angina which prevailed during successive years, from 1813 to 1816, in so many parts of the United States, adults, and those advanced in life, were the greatest sufferers. And although this may be looked upon as a striking departure of the disease from that class upon whom it is most predisposed to fall, and in whose systems it most delights to ravage, still there is something in even these instances of its prevalence, that creates doubts concerning its identity with a disease that observes no such pathological habitudes in regard to age.

3. Prominent among the pathological conditions of putrid sore-throat are the exudations of lymph, for not only in the fauces, but also in a number of cases this lymph makes its appearance during some period or other in the larynx or trachea, and thus becomes one of the most portending symptoms by which the disease can be characterized. These anatomical peculiarities, so far as the larynx and trachea are concerned, are said to be wanting in scarlet fever. Rayer says that although the exudations of lymph often extend to the lateral parts of the pharynx, and occasionally as far as the œsophagus, they are never observed after death in the larynx or trachea. The testimony of Dr. Tweedie is equally decisive on this point. In all the dissections he has made in scarlatina with anginose inflammation, no instance has occurred of finding in the larynx or trachea any membranous exudations. Has anybody ever observed a well-marked case of scarlatina terminate in croup?

4. Both diseases being characterized occasionally with an eruption, this, perhaps, if properly considered, may go to throw some light on the diagnosis of the two maladies. Scarlet fever of any variety, when it is marked by an eruption, preserves about the same degree of uniformity in the character of the eruption, as does either measles or small-pox. It is always the same eruption, varying, however, in hue and copiousness. Constantly, indeed, it consists in a diffused erythematous blush, and the lesions of the skin or efflorescence have always the appearance of not being raised above the cuticle. Whether the efflorescence be fiery or consist merely of dark purplish stains, the integrity of the phenomena mentioned above are preserved. If, now, we can place any reliance on the observations of our best authors on putrid sore-throat, the eruptions with which it has been characterized have had nothing like a uniformity of appearance. "Some," says Huxham, "had a "*cuticular* eruption, others had it of the *erysipelatous* kind; in others it was *pustular*." Ball speaks of the eruption as being *erysipelatous.* Thomas observed a dark-colored eruption, which he regarded as being unfavorable. Eberle says the eruption at first is *pale*, acquiring in most instances during the progress of the disease a *dark* or *livid* hue. These are examples sufficient to show that the alterations which take place in the skin preserve no identity of appearance; but, on the contrary, resemble those found in typhus fever fully as much as those peculiar to scarlet fever. This being the case we cannot see the propriety of classing putrid sore-throat with the exanthemata or eruptive fevers. Still further the reasons for this will diminish when it is recollected that the eruptions of putrid sore-throat are by no means of constant attendance in the epidemic visitations of the disease. In the Western Journal, (vol., 1) Dr. Warfield, of Paris, Ky., gives an account of an epidemic sore-throat which visited his neighborhood in 1821, in which not a single case was characterized with an eruption. Of the cases we have witnessed, none have been characterized with eruptions. Modern writers generally agree in the fact that the eruption is not at all of constant occurrence; and we know that this is, to a limited extent, the case with the proper eruptive fevers including scarlatina. But has ever any of the eruptive fevers been observed to prevail as epidemics *sine eruptione*, as we have just seen has been the case in regard to sore-throat?

5. Scarlatina, as well as measles and small-pox, by pretty general consent, destroys the susceptibility of the system to a second attack. Of the cases reported in this paper *four had been, two years previously to their illness with sore-throat, afflicted with scarlet fever*. These cases all belonged to the same family, all suffered their attacks of scarlatina at about the same time, and all were seized within a few days of each other with putrid sore-throat. I obtained the information of their having had the scarlet from the father, and from what I learned of him it was of the simple variety of the disease attended with a copious eruption and some distress of the throat. There are some pretty well authenticated instances on record where the same person has had scarlatina twice, (Beckner, Newman, Binns;) and Richter says that cases, not only of a second, but even of a third attack, have been noticed. Respectable as the testimony is to this point, we are nevertheless inclined to the opinions of Withering, Bateman, and Willan, who deny the possibility of a second attack. Cases we have witnessed ourselves, where, from some cause or other, the dregs of disease, left in the system, after a first attack, have continued, upon exposure to exciting causes, to revive some of the worst symptoms in the fauces for years afterwards, and it may be that it is these which have been mistaken for a secondary occurrence of the malady. Waiving, however, the further discussion of this question, we may remark that the instances of a secondary attack must be exceedingly rare; and this being the case it would be a very strange coincidence that four of such cases should take place in the same family, and all, too, at about the same time.

6. Most diseases attended with fever have something peculiar in the excitement with which they are characterized. In this respect synochus fever differs very widely from typhus. Great now, as all regard this difference, it is not more than equal to that observed between scarlatina proper and putrid sore throat. In the former the fever is generally of the synochus grade; in the latter it more nearly resembles typhus. Nor does the fact that scarlatina, once in a while, varies from this grade, becoming congestive or adynamic, alter at all the matter at issue, for we frequently see fevers of an inflammatory grade after going through the acute stages, from some peculiarity in the system or the circumstances with which the patient is surrounded, pass into a typhoid or putrid stage, that demands a total change in the treatment. Circumstances of this kind are of frequent occurrence in the experience of every physician; but no one because of this would regard the two diseases as being identical. Hence the excitement peculiar to sore throat may at any rate be regarded as being of sufficient importance to give it a respectable place among the diagnostic features of the two diseases.

7. Considered as an element of diagnosis, the effects of remedies may throw some light on the difference between these two diseases. Bloodletting has been regarded as a very available agent in most cases of scarlatina; particularly in those where the action of the heart and arteries is great. Most practitioners as well as writers speak of its effects in terms bordering on praise. This is not the case as it regards putrid sore throat. Here many writers, from Huxham down to our latest and best, unite upon the position that abstractions from the vital current are of doubtful propriety during even the first stage, while at any other period of the malady they manifestly do harm. In scarlet fever too, there is more or less tolerance to the loss of blood, when as a therapeutic agent it is but slightly indicated, while in putrid sore throat one bleeding often precipitates the patient into an irrevocable state of debility. Again the evacuant and depletive medicines so frequently resorted to in the treatment of scarlet fever find but little favor in sore throat. The best writers that we have consulted come to the conclusion that in sore throat sustaining and strengthening medicines to the general system are those upon which most reliance can be placed. These, indeed, as we shall see when we come to the treatment, are among the principal means by which the disease can be treated with any prospect of being subdued.

In concluding our remarks on this part of our subject, it may be submitted *in limine:*

1. That the two diseases seldom, if ever, prevail together, putrid sore throat being as liable to prevail with other epidemics as it is with scarlet fever.

2. That scarlet fever is confined pretty much to children, while putrid sore throat sometimes prevails as an epidemic among adults.

3. That the exudations of coagulable lymph, which we see in sore throat, extending, occasionally, to the larynx and trachea, producing *diptheritis* or secondary croup, is never found in these situations in scarlatina.

4. That the eruption in scarlet fever has a constant appearance, while in sore throat it varies, being, as often as any other way, *erysipelatous* or *pustular.*

5. That the diathesis is rather phlogistic in scarlatina; in sore throat it is the opposite, the former being attended with a synochus, the latter with a typhoid fever.

6. That medicines, depletive and evacuant succeed in scarlet fever, while in sore throat there is great necessity for sustaining and strengthening medicines.

Before leaving this division of our subject, we might say something on the question, of which we have already partially spoken, brought forward by M. Bretonneau. This learned pathologist, having witnessed an epidemic of sore throat, in which a large proportion of the cases were affected with that variety of disease called *angina membranacea;* and having found from the symptoms during the last stage, as well as from dissections, that a very prominent cause of mortality was false membranes in the air passages, concluded that the disease was identical with croup, *cynanche trachealis.* It constitutes no part of our present design to give this question a formal consideration. By the way, however, we may remark, that those who have practised extensively in sporadic croup, and noticed its etiology, course, and the effects of remedies upon it, will have use for but little from our pen in order to make up a decision. It might be said to those who have had but little experience in croup, that it has pretty high claims to the consideration of a distinct specific disease, having no symptoms nor anatomical characteristics in common with sore throat, further than the accretions of false membrane occasionally found in both diseases. But inflammation being the only known pathological condition necessary to the secretion of these heterologous productions, they may occur in any other disease, as well as in croup, where during the course of the disease the mucous membrane is either primarily or secondarily involved in this state. Hence the impropriety of making them pathognomonic, as the French author has done, in any disease. What is of occasional, he has insisted upon as being of constant, occurrence. But this would not hold good as it regards croup. Of all the cases of that disease which we have witnessed, three-fourths, we would suppose, were free from any morbid accretions in the larynx or trachea, the disease being spasmodic or inflammatory. This too, as we have already seen, has been the case in sore throat. Hence the presence of these false membranes is not necessary to the existence of either of the diseases; and we conclude that it would be about as reasonable to give remittent fever some new name or other, because it is occasionally complicated with visceral inflammation, as to give to putrid sore throat the name of *diptheritis* because it is occasionally attended with false membranes.

*Sequelæ.*—Without devoting any further remarks to diagnosis, we will now proceed to consider some of the conseqences entailed upon the system in cases where the disease has been of a malignant character, or where it has yielded imperfectly to the remedies addressed for its removal.

*Enlarged* or *hypertrophied tonsils*, we have witnessed oftener, than any other consequence of the malady. Without doubt this is owing to the conspicuous place which these glands have in the pathology. No case of any intensity takes place but what they are either highly inflamed, or are the seat of false membrane. Neither of these states, however, favors an enlargement of the tonsils so much as the ulcerative process with which they are occasionally affected. It is this process which seems to infuse the morbid growth upon which the enlargement depends. Once in a while, the glands become enlarged during the progress of ulceration; but most generally it is after the ulcers have imperfectly healed, when on slight exposures to cold or damp air, they again become inflamed, and after repeated attacks of this kind, they at last become permanently enlarged. The extent of the enlargement varies from the mere appearance, to a size sufficient to fill up almost entirely the lateral half arches of the palate. Among the various disturbances which accrue to the system from enlarged tonsils, none, perhaps, are more urgent, than the troublesome cough created, by which the stamina of the constitution is gradually undermined and the foundation sometimes laid for disease of the chest. Dysphagia also follows enlarged tonsils and makes it sometimes necessary for the patient to live entirely on fluids. These, in connection with the morbid secretions from the tonsils and the fauces, which are generally swallowed by children, and thus disorder the digestive aparatus, go to make a very grave complaint, and one to which medicines are applied, in the general, with but little success. Spontaneous mitigation, from a favorable set of circumstances, or extraordinary vigor of constitution, sometimes takes place, though this is a result of such seldom occurrence that it cannot be hoped for with any degree of confidence. Mention is made by authors of iodine, when the habit is scrofulous, of the chalybeate preparations, tepid salt baths, and of local applications of nitrate of silver, and butter of antimony, &c., all of which in their place may be tried; but we are satisfied, when the glands are considerably enlarged, that the most successful way of getting rid of them, is to invoke the aid of surgery. Ligature or excision, neither of which is attended with danger, will speedily dissipate the urgent symptoms and bring back the patient's health.

*Deafness* is frequently the result of enlarged tonsils. This depends most likely upon the inflammation or perhaps secretions of false membranes extending into the Eustachian tubes, by which their calibres become blocked up, and the transmission of sound is thus prevented. Accumulations of mucus, in these tubes, from those catarrhal affections that often follow attacks of sore throat, became, I have no doubt, an occasional cause of deafness, or impeded hearing. When the deafness depends upon the accumulations of mucus in the Eustachian tubes, emetics, or errhines, or indeed any thing that will excite sudden and violent motion in the parts, would seem to be indicated. Of the means for the relief of the more confirmed cases depending upon hypertrophy of the mucous lining of the tubes, or the secretions of false membrane within their calibre, we know nothing from experience; but in cases of this kind we are inclined to think favorably of the Eustachian catheter of Wathen, or the injection of compressed air as performed by Deleau.

*Thickened* and *nasal* speech are consequences that, once in a while, follow hypertrophy of the tonsils. When these take place, nothing short of excising the diseased glands will do much good, either in the way of palliation or cure.

Besides enlarged tonsils and their incidental consequences, we have sometimes left upon the fauces, after an attack of putrid sore throat, a species of chronic inflammation, or in some cases only a preternatural susceptibility of the mucous membrane in these parts to inflammation, which may be looked upon, as being a sequel of more frequent occurrence than enlarged tonsils. More than half of all the patients that we have had charge of, with putrid sore throat, were subsequently affected with this troublesome kind of inflammation. Slight catarrhal attacks operated very favorably to its supervention, though followed with equal facility in cases complicated with measles and pertussis. Anything, however, with some patients, even the slightest indispositions, were adequate to stir up an irritation in the fauces that would last for weeks. Mild cases yielded tolerably well to the common capsicum gargle. When this failed, muriatic acid and nitrate of silver were called into requisition. Temporarily subdued, as proved to be the case sometimes, even with these means, the disease would return to run its course again, and again subside upon the use of a similar prescription. Outraged, however, in the use of medicines to the parts, the physician at last will find a very available resource in scarifications of mucous membranes. I succeeded in one case by scarifying after some of the more potent agents of the materia medica had failed. This is most applicable to those cases attended with heat or burning sensations in the fauces. In such it produces prompt relief of the painful sensations; and thus the parts being to some extent relieved of the exciting cause of irritation, assume a better appearance, and seem much more amenable to the use of gargles.

These, in connection with the debilitated condition of the general system, and the depraved condition of the secretions, particularly of the mouth, which not unfrequently last for a long time after an attack of the malady, are only among the more urgent of the sequelæ. Others of a less frequent character, but sufficiently troublesome, have been met with, by those who have had much to do with the complaint.

*Duration.*—The length of time which putrid sore throat occupies in running its course varies according to circumstances. A good vigorous constitution, capable of resisting the putrid tendency of the disease confines the period of its possession of the system to its shortest limits. With individuals, particularly children of a lymphatic temperament or scrofulous diathesis, the disease delights to dwell. Lurking upon them in the form of diseased tonsils, or chronic inflammation or ulceration of the mucous membrane of the mouth, pharynx, larynx, trachea, œsophagus, it may last for a long time, to harrass and wear away the energies of the patient and foil the best directed efforts of the physician. Truly we may say, there is nothing more uncertain than the arrival of that period, at which there is, from all diseased action, a perfect exemption. Many cases get well in a week or ten days, while others, complicated with the affections, so prone to follow, may last for months. Excepting those cases, in which there is merely an inflammation of the fauces, we will find that the disease runs its course in those complicated with false membranes in the larynx or trachea in the shortest period of time. These cases, however, do not, so far as our experience is concerned, end in convalescence. Death is the usual result; and this happens after there is evidence of these membranes in the larynx or trachea, at about the same period, at which we find it to take place in croup, from the second to the fifth day. We are aware that Roche mentions a case of membranous inflammation of eight months duration; and Girauard a similar one that lasted two years; in both of which, the membrane was from time to time detached and replaced; but these are certainly, to say the least of them, rare cases, and of a character from which but little can be inferred. Properly speaking, they are anomalies, and hardly entitled to any consideration in making up an opinion on the duration of the malady.

*Termination.*—Under almost any circumstances, and with any plan of treatment, the mortality of putrid sore throat is immense. To the local affections is superadded a very malignant fever, either of which is sufficient to take life. Out of the twenty-two cases reported in this paper, nine died. As previously remarked, four of the cases which terminated fatally were of the membranous variety, and died with the symptoms of croup. With the rest the termination appeared to be the result of insidious anatomical alterations in the throat, that permitted the patients to appear as well as usual, until a very short period before their dissolution. The fatal event in these cases was not marked by any great suffering or violent agitation. One or two died seemingly without a struggle, when such an event by the friends was entirely unexpected.

Since I commenced writing this paper, I was called across the street to see a child two years old, that for a few weeks previously had appeared but slightly indisposed. On examination it was found to have sore throat, and notwithstanding means were vigorously applied, it died in less than forty-eight hours after my first visit.

My friend, Dr. Joshua Martin, of Xenia, who has been a reputable practitioner of medicine, for thirty years, in our county, (Green,) informed me that having witnessed more or less of the disease ever since he commenced practice, he had noticed that its progress and termination were not only exceedingly insidious, but that it was also characterized by high bills of mortality. My partner, Dr. Winans, who has been engaged in practice during a period of twenty-five years, gave me information of a similar purport. Called out to see a child of one of our citizens, he was asked to examine the fauces of another one that was sitting on the floor eating mush and milk. After making the examination, he told the friends that it would die in less than twelve hours. Distressing as the prediction was, it was verified.

Dr. Warfield, of whom mention has already been made, says that the disease in his vicinity (Paris, Ky.) progressed in a very '*deceitful*' manner, the patient retaining, in most cases, his muscular strength until within a few hours before his death. Nearly all of his cases, on the first appearance of the epidemic, died; which led to the conclusion among the people that the disease was incurable. From our own observations, therefore, and from what we have been enabled to learn from other physicians, the mortality in putrid sore throat falls but little short of what it is in malignant small-pox. The loss of thirty or forty cases out of every hundred, in either a sporadic or epidemic prevalence of the disorder, may be regarded as being not at all unusual.

*Prognosis.*—Anticipated as have been some of the principal features in the prognosis, it only remains for us to review the subject briefly, when we shall pass to a consideration of the treatment.

As we have seen, but little can be inferred from the manner in which the patient retains his muscular strength. This may leave him in any stage of the disease, or it may continue apparently but little impaired until within a few hours of his death. Nor can much be inferred from the character of the eruption, Dr. Thomas to the contrary notwithstanding; for as before remarked, this symptom is sometimes absent in every case of an epidemic, as was witnessed by Dr. Warfield when it prevailed in his vicinity; and when it does show itself its character is so various that there is nothing indicated upon which reliance can be placed. The condition, too, of the skin, as it regards moisture, is rather equivocal in its indications. Some patients pass to a fatal issue with an apparently normal condition of the skin, while others, in whom it seems dry, hot, and contracted, finally recover. A warm condition of the skin, if it is even attended with dryness, is certainly more favorable than where the temperature fails to reach the healthy standard, accompanied by a pale moist condition of the surface. From the condition of the circulation something more favorable may be augured. Fulness of the pulse, if it is even tolerably frequent, is the most favorable condition of the circulation; and when this takes place after it has been small, frequent, and irritable, it may safely be regarded as evidence of amendment. The local phenomena favorable to convalescence are a subsidence of the livid color of the fauces, abatement of the pain and swelling of the tonsils, uvula, and *velum palati*, and a casting off of the sloughs with facility, with a clean florid appearance of the parts on which they have been situated. The presence of these circumstances being connected with a good conditiotion of the respiratory functions, is well calculated to inspire hope.—Among the more obvious circumstances connected with an unfavorable termination, a lymphatic temperament, or a scrofulous, or tuberculous diathesis, stands prominent. Placed in a state of penury, with bad air, bad lodging, etc., but little can be predicted in favor of the patient's recovery. Much also will depend upon the extent of the disease in the throat. If this part in the early stages become livid and gangrenous, with extensive sloughing of the soft parts, there is no telling what will be the result. Abscesses may form of the character of retro-pharyngeal or retro-œsophageal, which proceeding very obscurely may take life without much warning. Hence in cases attended with dysphagia, dyspnœa, or putrid aphonia, we may look out for a probable connection of these symptoms with the pathological condition to which we have just alluded. But in the event that they are found not to depend upon such a grave lesion, their presence is still unfavorable. They perhaps depend upon extensive swelling of the soft parts in the throat, which is liable at any time to take life. The extension of the membranous inflammation to the larynx and trachea, perhaps, augur a fatal issue with most certainty. When decided symptoms of croup are once developed, we may reasonably conclude that '*the end is nigh;*' as we have not by observation, or the information we have received from other practising physicians, learned that any recoveries of consequence have taken place from this condition. Hemorrhage from the mouth or gums may be regarded as unfavorable. It denotes a bad condition of the circulating fluids; and on this account perhaps more than on any other, should its occurrence, at any time during the progress of the malady, be considered as a bad omen. Fothergill and Heberden think that the younger the patients are, the greater is their danger. This opinion, so far as our observations are concerned, is correct. Without doubt, the disease falls with greatest severity upon the young. In part, this may be owing to the fact that the disease often proceeds to an incurable stage before the necessity of properly attending to it is awakened; and also to another fact, that it is a very difficult matter in children to apply the necessary treatment to the parts locally diseased. But it must be confessed that there is something also in their organization that makes them more obnoxious to the remote cause than adults. A greater number of them contract it, when both are alike exposed to the sources of contagion.

*Treatment.*—Necessarily, the treatment proper for this malady, divides itself into *general* and *local.* To be correct it is indispensable that the general treatment be predicated upon proper views of the pathology. No one who has had any experience with the distemper, has failed to notice its prostrating tendency, and the singular ease with which cases, in the general, pass into a typhoid condition, bordering in some instances on putridity. This is strikingly seen in the manner in which some of the more vigorous and robust patients, as well as those of a worse class of habits, drop their accustomed energy of constitution, and become affected with a low grade of febrile excitement, and gangrenous ulcerations of the fauces. Huxham says he took some pains with the physicians in his part of England to make them understand the nature of the complaint, and distinguish it from other prevailing disorders to which it bore some degree of resemblance. He desired them to attend to "the sudden great dejection of spirits and strength," to the "small, quick, unequal fluttering pulse at the attack of *malignant squinsy.*" "Some," he continues, "were weak enough to tell me that the blood they had drawn was very *fine* and *rich*." But he adds, "Truly I found it as *lamb's blood* so *soft* and *loose* that you might cut it with a feather, giving off little or no serum." Other writers, notwithstanding some of them have recommended measures of depletion, in the commencement of the disease, have not failed to notice that such means were frequently accompanied by rapid prostration, and an aggravation of the graver symptoms of the malady. Boerhaave, Ball, Cullen, Thomas, and Eberle, have all hinted, some of them in pretty explicit terms, at these facts. Since, however, the group of symptoms which go to make up a case of putrid sore throat, has, by the refinements of medical nomenclature, been distributed under the terms stomatitis, tonsilitis, palatitis or uvulitis, we have been in danger of over-looking the most important feature of the disease: viz. its putrid tendency. Carefully bearing this in mind through every stage, the physician will do something more in the way of lessening the mortality to which the complaint is incident, than if he is indiscriminate in the selections of remedies suitable to such an important peculiarity. With some we know it to be a very difficult matter when they even see symptoms of debility, to imagine that they are real. Armstrong's '*congestions*' immediately take possession of the mind, and every thing connected with the condition is construed into an indication for depletion. The way must be opened for the imaginary suffocative excitement to pass out from the deep-seated organs of the body. Valuable as such doctrines are in a certain class of diseases, they will do no good in putrid sore throat. Nothing, indeed, will be found to be more fallacious, than the idea that the small, frequent, and irritable pulse here can be brought back to the normal standard by means that exercise an antiphlogistic influence upon the circulation. This brings us to a more special consideration of remedies, at the head of which, though perhaps not from its importance, stands

*Bloodletting.*—This has been among the remedies in this malady ever since it was first described. Huxham, as we have seen, regarded it as being of doubtful propriety. By his influence, as may be supposed, it held a minor rank with writers and practitioners generally, until the time of Armstrong, when, most likely, from his influence, it became entitled to more consideration. Accordingly we find it recommended by late writers in the first stage of the disease, as being a remedy of considerable value. Cases, in our opinion, might occur of a very sanguine or plethoric habit, where bloodletting would appear to be justifiable, or rather where it would do no harm. Such, nevertheless, are very rare, and when they do occur, surrounded by the best possible circumstances, more may be lost by premising the treatment with the abstractions of blood, than will be gained. In the sequel of the disease, we may need the vital influence of the blood drawn, to resist the force of chemical affinities, often so conspicuous in the fauces and other parts of the body. It should be recollected too that our patient is all the time bordering upon a state of asthenia, and whether precipitated into it by the morbid forces of the disease, or by the use of remedies, the result is the same. Hence it is that the lancet in this affection is amenable to objections founded upon pathological principles; and if used at all, this should only be after the nature of the case, and the circumstances surrounding it, have left no other alternative. In the membranous variety more than any other would its utility seem to be indicated. Here we have all the symptoms of primary croup, where as a remedy the lancet by common consent holds a reputation of the very first order. But not even here can much be said in its favor. The presence of this morbid production in the larynx or trachea, is associated commonly with other symptoms, relating to the general system, that go to create doubts concerning the propriety of decided measures of depletion.

*Emetics.*—Among all the remedies to which the practitioner has access, none will display better effects than emetics. Administered in the first stage, or during the period of incubation, they sometimes break up the train of morbid actions, and place the patient at cnce out of danger. At a later period they are well calulated to equalize the excitement, and restore the circulation to something like a healthy standard. The irritable, small pulse is more under the influence of emetics than any other remedy with which we are acquainted. The influence of emetics also in imparting to the skin a healthy diaphoresis and uniform temperature, is very obvious. Not less important are their local effects. The process of emesis, by equalizing excitement, possesses a singular efficacy in dispelling the pain, moderating the swelling, and allaying the local inflammatory excitement in the throat. Even when the parts here are ulcerous, attended with paroxysms of dyspnœa, or dysphagia, the operations of an emetic will generally give temporary relief. In children, emetics are almost our only means of cleansing the fauces from those foul, viscid and irritating accumulations that collect there in such great quantities. After vomiting they are usually better, and continue so until the parts become similarly affected. Concerning the efficacy of emetics in cases complicated with false membrane, our knowledge is limited. We have used them, in what cases we have witnessed, and always with more or less alleviation of the urgent symptoms; but we have had no case of this kind in which they, or any thing else, did more than palliate. Everything therefore considered, the efficacy of the emetics must strike every one at all acquainted with this affection as being beyond question. Without them, we know not how the treatment could, in any successful or satisfactory manner, be conducted. They are of use in every stage, and there are many indications presented, which they alone are calculated to fill. As it regards the particular emetic best suited to the complaint, those possessing stimulant effects are to be preferred. We have, nevertheless, when they seemed to be indicated, used tartar emetic and ipecac, with excellent results.

*Purgatives* are of some use throughout the whole course of the malady. But this is pretty much confined to keeping up the regular alvine evacuations, and working off the morbid secretions that collect in the supra, as well as infra-diapragmatic portions of the alimentary canal. For this purpose aloes, rhubarb, oleum ricini, &c., are much better than antiphlogistic purgatives. They accomplish the principal objects in view, without producing much incidental debility, an effect, as already observed, that should be avoided.

*Tonics.*—Much of the mortality in this affection being preceded by symptoms denoting an impoverished condition of the blood, and a lack of the vital energy necessary to keep the system generally and locally from passing into a putrid state, it would seem that sustaining and strengthening medicines are strongly indicated. What we know of their effects is decidedly favorable. An infusion of any of the vegetable tonics, such as cinchona, gentian, quassia, or columbo, or a proper preparation of some of the more permanent mineral tonics, may, as circumstances seem to justify, be used; and, if this is done with discrimination and judgment, they will prove to be important auxiliaries in the treatment. With the more stimulating corroborants, alcohol, opium, &c., we have no experience. From their prompt effects they might be found to be serviceable during the urgency of symptoms in the last stages. At any other period they occupy a position of less importance, than other articles which have been previously mentioned. We want something in this malady that will not only create, but that will keep up and permanently maintain a strengthening effect for a long time. The chalybeate preparations, on this account, as well as from the fact that they are the best calculated to alter the dissolved slimy appearance of the blood, are perhaps most valuable. This concludes what we have to say on means addressed to the general system. And although they may be found at variance with the teachings of some of our modern authors, we submit them, nevertheless, for consideration and trial; and with such tests of their accuracy we shall be satisfied.

*Local Remedies.*—Stress, not sufficiently great, or at all commensurate with its value, has been laid upon the local treatment. Upon the judicious selection and proper application of the local means, will very much depend the success of the practitioner. In vain will he combat general symptoms, if the throat externally and internally be neglected. Here the disease is seated, committing its ravages under the form of inflammation, gangrenous ulceraton, or the secretion of false membrane. Once securely situated here, this part becomes a focus of irritation, from which proceed morbific rays that extend to every part of the system; and hence in a great majority of the cases the malignity of the disease bears a direct proportion to the alterations which take place in the throat. Indeed I have never witnessed a fatal case without being able to trace the cause of it to the morbid anatomy of this part. A matter of great importance then, is to watch this part closely throughout the whole course of the malady, that we may give it the benefit of seasonable and vigorous measures of relief.

When the parts first become inflamed, it has been our practice to use the common capsicum gargle. This, in mild cases, where there is no very great lividness or abrasion of the stomato-pharyngean mucous membrane, will answer a good purpose. For a long time pepper has had a considerable reputation in affections of the throat. It was among the remedies of Hippocrates; and authors generally, from his day until the present, have spoken of it in very respectable terms. But its combination with salt and vinegar is a work of modern pharmacy, and does a great deal to enhance its usefulness. At any rate in all the conditions to which it is applicable, we prefer the compound preparation.

With evidence of mere distress in the fauces, the hydrochloric acid, either separately or combined with the tincture of capsicum, will be found to be a very available remedy. It is most applicable to those cases in which there is incipient gangrene, or a dark red appearance bordering on this condition. Nothing in our hands has checked this with so much facility as the hydrochloric acid. It corrects the offensive breath, the fetor arising from the parts, and serves as a detergent to remove the viscid irritating accumulations that always collect about the fauces when it is in a state of disease. Our mode of using it is in solution, combined with the tincture of capsicum, and of a strength, that, when used as a gargle, will not produce disorganization of the healthy mucous surfaces. With a preparation somewhat stronger than this, we paint the parts most diseased, from time to time, until the sloughs are cast off, and they assume the florid healthy color. Young children, we know from experience, are exceedingly averse to measures so irritating to the mouth and throat; and parents, we often find, weak enough to humor them, and forego a strict observance of the physician's directions. It should always, therefore, under such circumstances, be impressed upon the minds of parents, that an omission of duty on their part may lead to the loss of the child.

After the ulcerous process is once established, and the parts commence sloughing an irritating offensive sanies, *nitrate* of silver may be advantageously called to our aid. The great value of this salt in all similar affections, led Higgenbottom to apply it to ulcers so as to cause an eschar; and he asserts, that when this peels off, the sore beneath is found to be healed. Whether used to produce this effect or to change the morbid action, by which large portions of the soft palate are sometimes eaten off, the medicine cannot fail to be regarded with importance. But Mackenzie gives it a still higher claim to our consideration. He says that it is of great service in putting a stop to the fibrinous exudations that take place on the surface of the tonsils, and arches of the palate. Should this on extensive trial prove to be the case, the effect of the medicine as a prophylactic to the croup variety of the disease, in connection with its other acknowledged virtues, will go still further to enhance its value. Mackenzie's mode of using the medicine was in a solution, composed of a scruple of the nitrate to one ounce of water. Dr. Gibbs, (Braithwaite's Retrospect, from the American Journal of Medical Science, 1842), reports a case of cynanche maligna, followed by the symptoms of croup, in which a saturated solution of the nitrate of silver is said to have accomplished a cure. The case was that of a child four years old. On examining the fauces, an ulcer was seen, half an inch in diameter, over the left tonsil, and the other parts were covered with 'a thick, tenacious albuminous exudation.' The breathing was 'stridulous and very shrill,' with high fever, pulse 120, and thirst. Calomel, antimonial powder and ipecac. had previously been used, but it was thought without any benefit. The sulphate of copper was then administered until it produced emesis and brought away 'shreds of membranous matter.' After this a saturated solution of the nitrate of silver was applied with a sponge to the ulcer and fauces, thrusting it far back into the pharynx. Every application was followed by relief until the cure was accomplished. We differ from Dr. Gibbs, in ascribing entirely to the nitrate the happy issue of this case. '*Shreds*' of the false membrane, it seems, were brought away by the process of emesis, before the nitrate was applied. The nitrate of silver no doubt exercised a favorable influence over the ulcerous process present, and to this feature of the case we believe its usefulness was pretty much confined.

Recently *alum* (sulphate of alumina and potassa) has been brought forward, and we may say extolled by Velpeau, of Paris, as a remedy in every stage of sore throat. This distinguished author derived his first knowledge of the efficacy of alum from his preceptor, Bretonneau, who used it while in the hospital of Tours, with great success. He also cites the experience of Laennec, of Nantes, in its favor. Velpeau used it where the throat was red and painful, the tongue foul, the amygdalæ much tumefied, with great difficulty of swallowing and breathing; and in two cases where suppuration appeared to be imminent. The result was always the same, "the pain and febrile symptoms almost always diminish in an evident manner after the first or second application, and the patient improves so rapidly that on the third or fourth day (and in some instances on the following day) he feels himself perfectly well." His manner of using it was either in gargle or in powder. The powder was resorted to only in severe cases, and was applied to the parts with the tip of the finger. High and imposing as the authority is from which we derive this testimony, we are inclined to think it too positive and general to stand the test of experiment. We should have expected *a priori*, that alum possessed considerable virtue in diseases of the throat; but to suppose it applicable to every form of disease here—simple inflammation, membranous inflammation, and the various stages of ulceration, makes rather too strong a draft on credulity for our purpose. Medicines in a general way possess an efficacy for some particular feature or phase in the disease to which they are applied. This doubtless will be found to be the case with alum. And, if the encomiums to which we have alluded shall serve no other purpose, they may lead to such experiments with the drug as will develope the particular form of the disease in which it is beneficial.

From its acknowledged properties as a stimulant, antiseptic, etc., *creasote* should hold a respectable place among the remedies in sore throat. So far though as we are acquainted, the trials of its efficacy are not sufficiently numerous to give it anything like a decided reputation. Its use of course would be contraindicated in cases where the inflammation in the fauces was at all acute. But when the parts are affected with such a low grade of action that the vital resistance is no longer adequate to resist the play of chemical affinities, and gangrene as a consequence results, the creasote from its several properties, stimulant, antiseptic, and escharotic, would seem to be pretty fairly indicated.

Towards *scarifications*, when the fauces are dry, glossy, and attended with burning sensations, that, in the language of the older writers, denote great '*defluxions*,' we have considerable partiality. When the parts are well scarified, and bled freely, the urgent symptoms usually subside, and they then become more amenable to other remedies. It should be observed that scarifications will only prove serviceable in cases where there is considerable arterial excitement in the parts, and this is indicated by the burning pain. In other conditions, as for instance where the parts are gangrenous, they might prove injurious.

External applications are in many instances necessary for the tumefaction of the glands on the side of the neck. Usually the parotid, sub-maxillary, and in many cases, the lymphatic glands, are considerably enlarged. Sometimes it is necessary to promote suppuration in order to relieve the internal engorgements about the fauces. For this pupose, emollient poultices should be applied. We think much more of those than of liniments. They combine heat and moisture, two valuable agents in the accomplishment of our object. When counterirritation is indicated, blisters and sinapisms have been the principal agents upon which we have relied. It should be borne in mind, however, that the inflammations, either externally or internally, which usually occur in putrid sore throat, are much less under the influence of counterirritation than we commonly find to be the case with those connected with other diseases.

To the general surface, when it is found to be unequal in its temperature, pale, and covered in places with a clammy perspirable matter, we may apply tepid vinegar and water until the parts are sufficiently cleansed, after which frictions with flannel should be instituted, until the surface puts on a florid appearance. Measures of this kind, by maintaining an equilibrium of temperature, will promote healthy diaphoresis, and invite towards the surface, congestions that would otherwise accumulate upon the deeper-seated organs of the body.

Before concluding the treatment, it might perhaps be proper to say something on the regimen to be observed. Commonly the appetite is poor and the stomach weak. But we feel assured, that articles calculated, to a reasonable extent, to strengthen the one and excite the other, will do more good than if they are light and scanty. We need the influence of a generous nourishing diet—something that will keep the functions of nutrition in vigorous play, during the whole course of the disease. Beef-tea, potatoe soup, and milk porridge, with wine occasionally, will answer very well, and perhaps do all that can be done, in this way, to obviate the septic tendency to which the complaint is liable.

The principal points upon which the attention should be fixed in the treatment, may now be summed up in the following propositions:

1. The disease, in the general, being characterized by a low grade of morbid actions, is not, as a consequence, amenable to decided antiphlogistic remedies.

2. The *local* treatment is of as much, if not of more importance, than the general.

3. The resources of the system should be carefully economized throughout the whole course of the disease; and all pains taken with strengthening medicines, and regimen, to avoid the stage of prostration.

Jamestown, Ohio, Dec. 7, 1844.

